# Analysis of the Low-Carbon Transition Effect and Development Pattern of Green Credit for Prefecture-Level Cities in the Yellow River Basin

**DOI:** 10.3390/ijerph20054658

**Published:** 2023-03-06

**Authors:** Jingcheng Li, Menggang Li, Tianyang Wang, Xiuqin Feng

**Affiliations:** 1 School of Economics and Management, Beijing Jiaotong University, Beijing 100044, China; 2 Beijing Laboratory of National Economic Security Early-Warning Engineering, Beijing Jiaotong University, Beijing 100044, China; 3 School of Marxism, Shandong University of Science and Technology, Qingdao 266000, China

**Keywords:** green credit, carbon emissions, the Yellow River Basin, sustainability

## Abstract

Green credit is a vital instrument for promoting low-carbon transition. However, designing a reasonable development pattern and efficiently allocating limited resources has become a challenge for developing countries. The Yellow River Basin, a critical component of the low-carbon transition in China, is still in the early stages of green credit development. Most cities in this region lack green credit development plans that suit their economic conditions. This study examined the impact of green credit on carbon emission intensity and utilized a k-means clustering algorithm to categorize the green credit development patterns of 98 prefecture-level cities in the Yellow River Basin based on four static indicators and four dynamic indicators. Regression results based on city-level panel data from 2006 to 2020 demonstrated that the development of green credit in the Yellow River Basin can effectively reduce local carbon emission intensity and promote low-carbon transition. We classified the development patterns of green credit in the Yellow River Basin into five types: mechanism construction, product innovation, consumer business expansion, rapid growth, and stable growth. Moreover, we have put forward specific policy suggestions for cities with different development patterns. The design process of this green credit development patterns is characterized by its ability to achieve meaningful outcomes while relying on fewer numbers of indicators. Furthermore, this approach boasts a significant degree of explanatory power, which may assist policy makers in comprehending the underlying mechanisms of regional low-carbon governance. Our findings provide a new perspective for the study of sustainable finance.

## 1. Introduction

By the end of 2021, China’s green loan balance had reached a world-leading $2.5 trillion, and it continues to grow [1]. As an essential element of China’s sustainable financial system, green credit comprises a collection of policies and institutional frameworks that leverage credit to encourage businesses to engage in energy conservation and reduce their carbon footprint. In practice, banks may elect to withhold loans from corporations that fail to meet established emissions standards, thus reinforcing the importance of environmentally responsible behavior in the business community [2].

Green credit has emerged as a pivotal tool for China in its efforts to meet its ambitious carbon control targets, providing crucial support for low-carbon transition projects throughout the country. Similar to the Ganges Basin in India [3,4] and the Nile and Tana basin in Africa [5,6], the Yellow River Basin has historically relied on unsustainable approaches in its agricultural and industrial production activities. As a result, the region now confronts significant obstacles in its low-carbon transition efforts that are compounded by issues of water scarcity and ecological fragility. The Yellow River Basin is an important ecological barrier and an ecologically fragile area in Northern China. Due to resource depletion and de-industrialization, numerous cities in the Yellow River Basin are currently unable to complete low-carbon transition. Thus, it is necessary to support low-carbon transition in such cities through the rational allocation of credit resources (green credit) [7].

**Figure 1:**
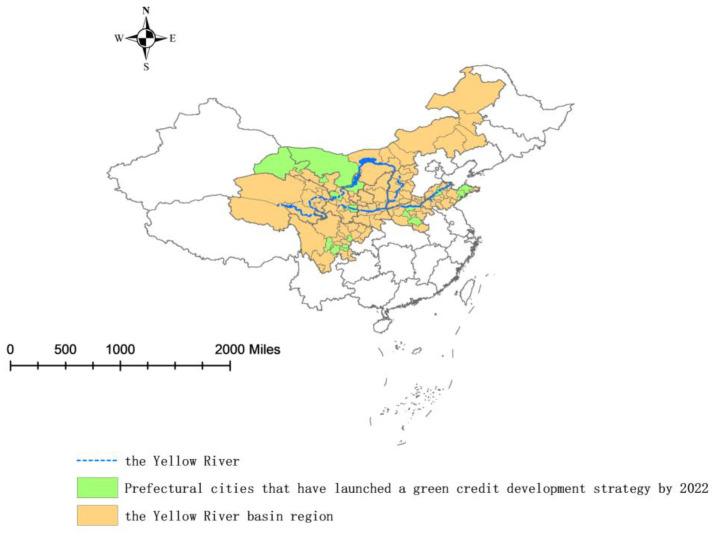
Green credit development in the Yellow River Basin.

Green credit and other sustainable financial products have been successfully implemented in developed countries [8,9]. Evidence from China also suggests that green credit can enhance energy efficiency, though this effect is more pronounced in the eastern region and less pronounced in the central and western regions [10]. Although there are an increasing number of studies on the impact of green credit on low-carbon transition [7,11,12], comprehensive assessments of green credit’s role in the low-carbon transition of the Yellow River Basin are scarce. This has resulted in slow progress in green credit development in the region, with most prefectural-level cities yet to devise suitable green credit development patterns (Figure 1), which may delay China’s carbon peak and carbon neutralization plans [13].

**Figure 2:**
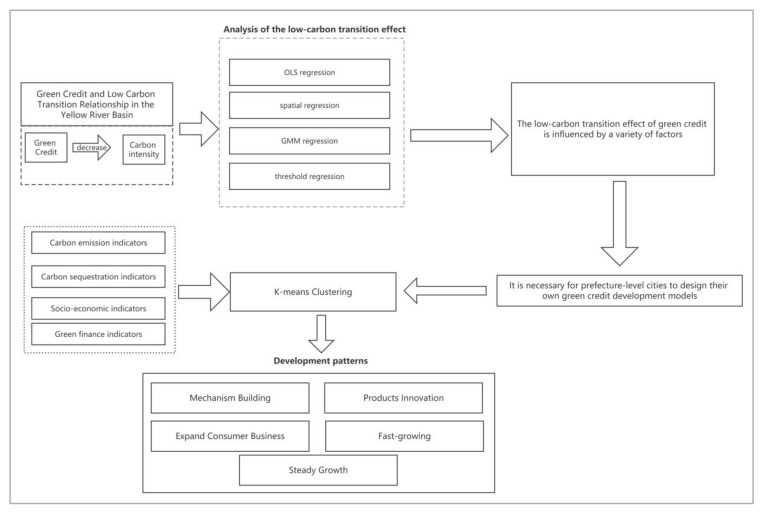
Research framework.

This paper was aimed to analyze the influence of the green credit scale on carbon emission intensity in the Yellow River Basin and to identify potential factors affecting the role of green credit in low-carbon transformation through heterogeneity analysis. This will provide a basis for selecting an appropriate green credit development pattern to facilitate green transformation in the Yellow River Basin and offer useful experiences for other developing countries in environmentally friendly governance. Figure 2 illustrates the research framework of this paper.

The main contributions of this paper are as follows: First, we investigated the relationship between green credit and carbon emission intensity using data from prefecture-level cities in the Yellow River Basin. According to our empirical results, green credit would be supported as a measure for the low-carbon transition in the Yellow River Basin. Second, in order to address the practical issue of how to differentiate the development of green credit policies, a clustering algorithm (k-means) was used to classify the green credit development plans of 98 prefecture-level cities in the Yellow River Basin.

This paper is organized as follows. The Section 2 reviews the relevant literature. The Section 3 outlines the data sources, empirical methods, and variable settings used. The Section 4 presents the results of the regression and clustering analyses. The Section 5 provides specific recommendations for the development of green credit in each category of city based on a discussion of the empirical results, and the Section 6 summarizes the main conclusions of the study.

## 2. Literature Review

Banking and finance play fundamental roles in public policy and economic performance, as well as in all forms of commerce and industry, and adequate financial support is also essential for the sustainable development of society [14]. As far as we know, the term “green credit” mainly appears in research related to China. In Western countries, scholars’ research on environmental finance and sustainable finance is highly similar to China’s green credit research. In 1998, Salazar wrote that financial institutions could promote capital investment in low-carbon environmental industries through “green credit” [15].

The theoretical model between financial support and sustainable development was first proposed by Bovenberg [16] and has since been further developed by subsequent scholars [17,18,19]. In brief, sustainable financial products such as green credit can influence regional low-carbon transitions through three theoretical channels: Firstly, financial institutions can use green credit to raise the credit threshold, which can curb the excessive development of highly dyed industries and avoid the severity of environmental pollution problems, thus achieving the harmonious development of economy and environment. Secondly, providing financial credit support to green industries, ecological industries and recycling industries can promote economic structure upgrading and low-carbon transformation. Finally, green credit requires improved transparency regarding enterprises' environmental information, which can lead to targeted governmental control and targeted social supervision to guide enterprises and social production and lifestyle changes. Thus, we put forward the following hypothesis:

**Hypothesis** **1.** *The increase in the scale of green credit in the Yellow River Basin region will help promote the local low-carbon transition and reduce carbon emission intensity*.

Sustainable finance is seen as helping developing countries achieve their low-carbon transition by making up for the inadequate enforcement of environmental regulations [20]. However, evidence from Pakistan, Poland and Africa shows that financial development does not necessarily lead to the improvements in sustainable development capacity [21,22,23]. The reason for this phenomenon is that the process of low-carbon transformation is affected by various factors. Table 1 shows the main influencing factors.

The Yellow River, with a total length of 5464 km, originates from the Tibetan Plateau and enters the sea in Shandong Province, passing through nine different provincial administrations; it shows strong heterogeneity in economic development levels, natural resources, population size and cultural traditions in the upper, middle and lower reaches [32]. Thus, we put forward the following hypothesis:

**Hypothesis** **2.** *There is regional heterogeneity in the low-carbon transition effect of green credit in the Yellow River Basin*.

Recent evidence indicates that there is a long-term bivariate positive correlation between urbanization, energy consumption and carbon emissions [33,34], the significance of which varies by province depending on the size of the economy [35]. In this context, we put forward the following hypothesis:

**Hypothesis** **3.** *The level of urbanization may be a threshold variable affecting the effect of green credit’s low-carbon transition*.

Rice and Strahan argued that competition in the banking industry can make allocation more efficient [36]. Evidence from France suggests that a decline in bank competition can dampen credit activity [37]. As the main implementer of green credit, the role played by banks and other financial institutions in the green transition is crucial. Competition will motivate banks to launch more attractive green credit operations, which will consequently promote the low-carbon transformation role of green credit [38]. In this context, we put forward the following hypothesis:

**Hypothesis** **4.** *The level of bank competition may be a threshold variable affecting the effect of green credit’s low-carbon transition*.

Although there have been relatively few studies on sustainable financial development patterns, scholars generally agree that there should be a variety of sustainable financial development patterns. Schoenmaker thought that the development framework for sustainable finance needs to include economic, social, and environmental dimensions, and the development of green credit requires an appropriate mix of all three dimensions [39]. Ziolo et al. set 15 sustainable development goals based on the current status of sustainable finance in the EU countries belonging to the OECD [40]. In 2019, a comprehensive indicator evaluation system was designed by Ziolo et al. to measure the sustainable financial development prospects of European countries that included 12 economic indicators, 28 social indicators, 7 environmental indicators, and 16 sustainable financial indicators [41]. Rebai et al. argued that the concepts of corporate social responsibility, socially responsible investment, ethical investment, and cleaner production need to be added to the development connotations of sustainable finance [42]. Mazzucato and Semieniuk built a sustainable financial risk index system that included 11 low-carbon transition projects and 11 financial participants to map out how finance can influence the directionality of innovation in the future [43]. Table 2 shows the main literature on the studied subjects and the involved variables.

Given that the existing literature supports a diversity of sustainable financial development patterns, we put forward the following hypothesis:

**Hypothesis** **5.** *Green credit development patterns in the Yellow River Basin should be diverse*.

Despite the growing significance of green credit in promoting a low-carbon transition in China, the literature has paid scant attention to the Yellow River Basin, and empirical investigations of the heterogeneity and threshold effects of green credit in the region are notably absent. As a result, there is a dearth of sufficient empirical evidence to inform local green credit development in the area. Furthermore, the design of sustainable financial development patterns in foreign countries often relies on a multitude of indicators that are generally tailored to the well-established statistical systems of developed nations. As a result, such patterns may not be readily applicable to China and other developing countries, as they require vast quantities of data that may be unavailable. Additionally, the complexity of the indicator settings raises the threshold for policymakers attempting to implement these patterns.

In order to fill in the above research gaps, this paper makes the following contributions. Firstly, based on the panel data of 98 prefecture-level cities in the Yellow River Basin from 2006 to 2020, this study comprehensively examined the association between green credit and local low-carbon transition. Secondly, in order to make the indicator setting of green credit development patterns more concise while ensuring explanatory strength, this study introduced a k-means clustering algorithm and then divided the green credit development patterns in the Yellow River Basin into five categories, thus expanding the application scenarios of the clustering algorithm.

## 3. Materials and Methods

### 3.1. Study Area and Data Source

After excluding the 12 cities that lacked the necessary data (Gannan Tibetan Autonomous Prefecture, Linxia Hui Autonomous Prefecture in Gansu Province, Aba Tibetan Qiang Autonomous Prefecture in Sichuan Province, Haibei Tibetan Autonomous Prefecture in Qinghai Province, Huangnan Tibetan Autonomous Prefecture, Hainan Tibetan Autonomous Prefecture, Guoluo Tibetan Autonomous Prefecture, Yushu Tibetan Autonomous Prefecture, Haixi Mongolian and Tibetan Autonomous Prefecture, and Alxa League in Inner Mongolia, Laiwu, and Baiyin), this study obtained panel data on 98 prefecture-level cities in the Yellow River basin for the period from 2006 to 2020.

Since 2010, an increasing number of scholars have combined satellite data with a terrestrial carbon model to obtain more accurate carbon emission data [44]. For carbon emission and carbon sequestration data, we followed the study of Chen et al. [45], which measured carbon emission and carbon sequestration amounts based on satellite remote sensing data. We replaced missing data using local energy consumption, forest cover, urban green area change trends and the CEADs database [46]. Therefore, this study avoided the errors caused by the traditional IPCC coefficient method.

We mainly obtained the economic and social indicators through provincial statistical yearbooks [47], city statistical yearbooks [48] and the CSMAR dataset [49]. Some cities did not publish economic data for 2017, and we used interpolation to fill in the missing data. To construct indicators that reflect the intensity of environmental regulation, we collected government work reports for each year during the sample period of each city and conducted textual analysis. To construct the green development trend of each city, we collected the major media reports related to green innovation in each city.

### 3.2. Regression Model and Variables Settings

To investigate the correlation between green credit and low-carbon transition in the Yellow River Basin, we designed the regression model expressed as Equation (1):

(1) $$Carbon=\beta_{0}+\beta_{2}GreenC+\theta_{2}Controls+\delta_{i}+\gamma_{t}+\varepsilon_{i,t}$$

where Carbon means the carbon intensity, GreenC means the green credit policy, Controls means a series of control variables, $\delta_{i}$ means the individual effect, $\gamma_{t}$ stands for the year effect, and $\varepsilon_{i,t}$ means the residual errors. Table 3 shows the details of each variable involved in the regression.

Explanatory variables: green credit level (GreenC). Following the method of Zhang et al. [50] and Hu et al. [51], we used the difference between the size of all loans minus the size of loans to highly polluting industries to represent the level of green credit.

Explained variable: carbon emission intensity (Carbon). There are several variables that measure the low-carbon transition process; we chose carbon intensity because it is more intuitive and concise to calculate and because it has been used by the Chinese government to set the “carbon control” goals [52].

Control variables: based on previous research [24,25,26,27,28,29,30,31], we chose environmental protection (EP), the number of residents (PEO), the education level (EL), the industrial structure, the degree of openness (FDI), and the economic potential (Growth) as control variables.

The statistical description of the variables is shown in Table 4.

### 3.3. K-Means Clustering

As a technical approach to data mining, clustering has been widely used in region classification and decision making because machine learning algorithms are sensitive to the associations among indicators. There are many different branches of cluster analysis algorithms, including hierarchical clustering, fuzzy clustering, systematic clustering, and k-means clustering.

K-means clustering is a method of vector quantization, originally derived from signal processing, that aims to partition n observations into k clusters in which each observation belongs to the cluster with the nearest mean (cluster center or cluster centroid), serving as a prototype of the cluster [53]. K-means clustering minimizes within-cluster variances (squared Euclidean distances). The k-means clustering algorithm was chosen because of the strong explanatory power of this method and its ability to establish correlations between clustering results and each indicator, which consequently enhances the credibility of clustering results and understandability. Due to these advantages, the application of k-means in the field of economic management has started to increase in recent years [54].

In this study, we standardized all clusters to build a sample collection $X=\left\{x_{1},x_{2},\cdots ,x_{n}\right\}$. Then, k sample points were randomly selected as the initial clustering centers, the distance from each sample to the class center was calculated, and each sample was assigned to the nearest central class, which constituted the initial clustering result. Then, the mean value of the samples in each class was calculated as the new class center, and the above steps were repeated until convergence. In this study, we used squared Euclidean distance $d\left(x_{i},x_{j}\right)$ to represent the distance or similarity between samples.

(2) $$d\left(x_{i},x_{j}\right)=\sum_{k=1}^{m}{\left(x_{\mathrm{ki}}-x_{\mathrm{kj}}\right)}^{2}=\sum_{k=1}^{m}∥x_{\mathrm{ki}}-x_{k}{∥}^{2}.$$

(3) $$\mathrm{WCSS}=\sum_{l=1}^{k}\sum_{C\left(i\right)=l}∥x_{i}\;-\;{\bar{x}}_{l}{∥}^{2}.$$

The convergence condition or evaluation index of a clustering algorithm is the sum of the distances between the samples and the centers of the classes they belong to. In Equation (2), the sum of the distances $d\left(x_{i},x_{j}\right)$ is WCSS. The smaller this index is, the greater the similarity between similar classes and the better the clustering effect; see Equation (3). The degree of distortion decreases as the category increases, and if the degree of distortion is greatly reduced when a critical point is reached, it slowly decreases afterward. This critical point can be considered the point with better clustering performance, and its image resembles an elbow, so the process is named the elbow method.

The elbow method can determine the optimal number of groups based on the sum of squares of deviations under different group numbers [55]. Therefore, using this method to determine the number of clusters could improve the objectivity of classification [56]. When the inflection point of an image is not obvious, judging the number of clusters by subjective means often leads to errors. To enhance the robustness of clustering, this study also applied the method of Mohajer et al. [57] to avoid the overestimation of the number of clusters.

In order to reflect the green credit development needs and development status of different cities in the Yellow River Basin, we constructed a comprehensive index system that combines the “double carbon” target with the local economic and social development level. More specifically, 4 static indicators and 4 dynamic indicators were selected to cluster the 98 cities in the Yellow River Basin. In order to illustrate the cross-sectional characteristics of carbon emissions in various types of cities, static indicators were chosen based on the findings of prior studies. Dynamic indicators can track the dynamic patterns of green finance development and carbon reduction trends of various cities. Table 5 displays specifics for these 8 indicators.

The main challenge of energy saving and emission reduction in the Yellow River Basin at present is balancing economic development and carbon emission control. Therefore, we selected GDP per capita, economic growth, and carbon emissions to reflect such dynamic relationships.

Furthermore, due to the central government’s promotion, the achievements of energy conservation and emission reduction efforts are now closely linked to the performance of officials, who use the media to publicize the importance they attach to green development. Through the data-mining of keywords such as “green credit”, “green insurance”, “green bonds”, and “carbon trading”, we obtained the number of reports related to green finance development in each city from Chinese mainstream media (People’s Daily, Guangming Daily, Economic Daily, and Sina Finance) to reflect the degree of green finance development in different cities.

The industrial structure change was chosen to reflect the process of industrial upgrading. As a traditional heavy industrial region, developing high value-added tertiary industries is an effective path for the Yellow River Basin to embrace carbon peaking. As China’s population will slowly decline in the future [58], a city’s population could reflect the attractiveness of its industries and its future competitiveness. We chose the average change in the residents’ numbers to reflect the development potential.

We also chose the level of carbon emissions per capita as an indicator to reflect the resistance to emission reduction. If a city has a high level of carbon emissions per capita, the employment and livelihood of local residents have become dependent on high carbon emissions. Compared with previous studies, we added carbon sequestration growth as a dynamic indicator to reflect land use, land-use change, and forestry (LULUCF) activities. Thus, the clustering analysis of green finance development was more accurate [59].

## 4. Results

### 4.1. Benchmark Regression Results

To test the Hypothesis 1, we chose the level of green credit development as the independent variable and the local carbon emission intensity as the dependent variable, and then we conducted a regression analysis based on the panel data of 98 prefecture-level cities from 2006 to 2020.

Hausman test results showed that the fixed effect model was better than the random effect model for model estimation. Table 6 provides the regression results. Columns (a), (b) and (c) represent Equation (1) with different numbers of control variables. As the number of control variables rose, the R^2^ increased from 0.19 to 0.96. Including time and individual effects, the coefficient of green credit (GC) shown in column (c) was −0.040 and passed the 10% significance level. The regression results clearly showed that the development of green credits in the Yellow River Basin effectively reduces the carbon intensity of the region and contributes to low-carbon transition. These findings support Hypothesis 1.

### 4.2. Endogenous Problem

Missing variables or green credit measurement errors could have caused endogenous problems in the model. The GMM (Generalized Method of Moments) model is a widely used estimation method in econometrics. The basic idea of this method is to use sample moments to estimate population moments and minimize the difference between the estimated population moments and the sample moments to obtain consistent parameter estimates. The advantage of the GMM method is that it can handle endogeneity issues, i.e., the correlation between the independent variables and the error term, and it has strong empirical properties.

We used the GMM model to mitigate the potential statistical errors associated with adopting the first-order lag term of green credit as an instrumental variable (IV). The estimation results generated by the GMM model reported in Table 7 (−0.338) were consistent with the benchmark regression results.

### 4.3. Spatial Spillover

To further enhance the robustness of the regression results, we used a spatial regression model to incorporate spatial spillover effects into the regression, and the results of the LM and Hausman tests supported our use of a spatial Durbin model with double fixed effects [60].

(4) $$\;Carbon_{it}=\delta_{0}+\eta_{1}\;Carbon_{i,t-1}+\rho_{1}\overset{n}{\underset{i=1}{\sum }}w_{ij}\;Carbon_{jt}+\rho_{2}\overset{n}{\underset{i=1}{\sum }}w_{ij}\;GreenC_{jt}+\rho_{3}\overset{n}{\underset{i=1}{\sum }}w_{ij}\;Controls+\delta_{1}\;GreenC_{it}+\zeta_{it}\;$$

In Equation (4), i denotes the region, t denotes e year, and $w_{ij}$ denotes the spatial proximity of region i and region j. A geographic distance weight matrix is used in this part.

The results of this spatial Durbin model are shown in Table 7. The coefficient of GreenC was still negative (−0.067) and significant at the 5% significance level.

### 4.4. Robustness Check

The robustness of the basic regression results was checked by replacing the explanatory variables, adding control variables, and testing the effect of policies. First, we replaced the explanatory variable of carbon intensity with energy intensity in the column. Second, we expanded the number of control variables by adding the number of Internet users to the list of control variables to reflect the progress of digitalization. Third, as the Chinese government officially launched the green credit system in 2012, we selected 2012 as the time point of policy shocks to examine the association between declining carbon intensity and the release of important policies for green credit. The variable GreenC was set to 0 before 2012 and 1 after 2012 to conduct the regression of Equation (1).

As seen in Table 7, the results of the robustness check showed that a 1% increase in the size of green credits was expected to result in a 0.37% decrease in energy intensity. This finding suggests that green credits have a positive effect on energy efficiency and emission reduction, which consequently increases the credibility of green credits’ ability to reduce carbon intensity.

The coefficient of green credit on carbon emission intensity remained negative (−0.041) and significant at the 10% level after the inclusion of the control variable representing the digitization process. It is worth noting that the number of Internet users had a significant suppressive effect on carbon intensity, with a coefficient of −0.028 that was significant at the 5% level. These findings support the contribution of digitalization to low-carbon transition [61].

### 4.5. Heterogeneity Analysis

#### 4.5.1. Regional Heterogeneity

As previously mentioned, the significant environmental disparities across the upper, middle, and lower reaches of the Yellow River Basin may have resulted in heterogeneity in the correlation between green credit and low-carbon transition. To this end, we set the following delimitation criteria: upstream (including Qinghai, Sichuan, Ningxia, and Gansu), midstream (including Shaanxi, Shanxi, and Inner Mongolia), and lower stream (including Shandong and Henan). It is worth noting that traditional classification places Inner Mongolia Autonomous Region in the upper reaches of the Yellow River. However, based on the sample of this study, most Inner Mongolia cities fall into the middle reaches of the Yellow River, so we classified Inner Mongolia as part of the middle reaches for our purposes.

Table 8 displays the results of the regression analysis. The findings indicate that the impact of green credit on carbon reduction varies across regions, with a higher efficiency observed in the lower reaches (−0.376), possibly due to the region’s higher level of financial development. These results support Hypothesis 2 and highlight the importance of tailoring the development of green credit to local conditions in the Yellow River Basin.

#### 4.5.2. Threshold Heterogeneity

The objective of this section is to investigate the impact of urbanization rate and bank competition level on the effectiveness of green credit on promoting low-carbon transition. To achieve this, we employed a threshold regression model. Following the approach of Kremer et al. [62], we established a dynamic threshold model in Equation (5):

(5) $$\begin{array}{c} {\;\mathrm{Per}\;}_{i,t}=\alpha_{0}+\alpha_{1}{\;\mathrm{Per}\;}_{i,t-1}+\beta_{1}{\;\mathrm{GreenC}\;}_{i,t}I\left(q_{i,t}⩽\gamma \right)+\\ \beta_{2}{\;\mathrm{GreenC}\;}_{i,t}I\left(q_{i,t}>\gamma \right)+\sum \gamma_{j}{\;\mathrm{Control}\;}_{i,t}^{j}+\theta_{i}+\varepsilon_{i,t} \end{array}$$

where $q_{i,t}$ denotes the threshold variable (urbanization level and financial competition level), $I\;\left(\cdot \right)$ is the indicative function, and γ is the specific threshold value. The degree of local bank competition was measured using the market shares of the five largest state-owned banks, namely the Bank of China, Industrial and Commercial Bank of China, China Construction Bank, Agricultural Bank of China, and Bank of Communications, represented as the CR5 indicator. A higher CR5 value implies a lower degree of local competition.

**Figure 3:**
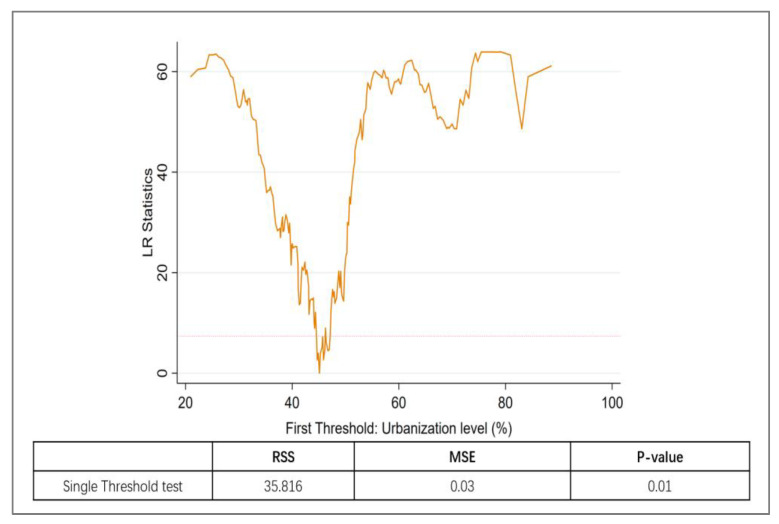
Threshold test for urbanization level.

**Figure 4:**
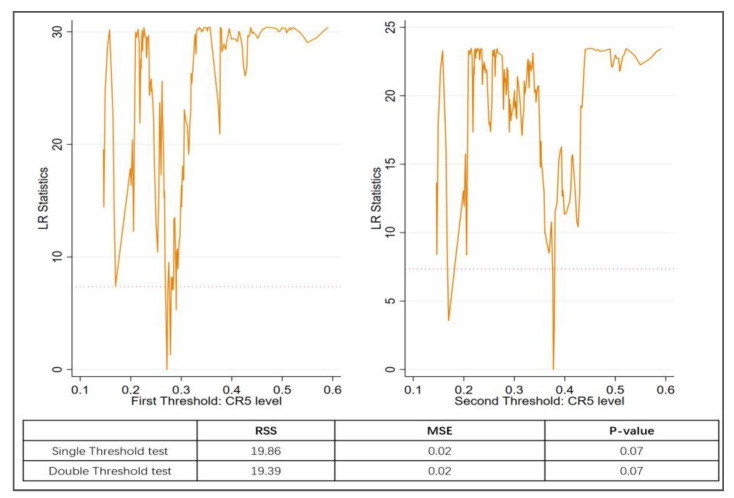
Threshold test for bank competition level.

The results of the threshold effect test for urbanization are shown in Figure 3. Based on the *p*-value in Figure 3, a single threshold value (45.1%) was identified for the level of urbanization that affects the inhibitory effect of green credits on carbon intensity. Figure 4 shows a double threshold phenomenon for the degree of competition in the financial market, with the first threshold being 0.272 and the second threshold being 0.377.

The results of the threshold effect test for bank competition are shown in Figure 4. Cities with an urbanization level higher than 45.1% were classified as having high urbanization, whereas those with an urbanization level lower than 45.1% were considered to have low urbanization. Regarding the degree of bank competition level, cities with a CR5 below 0.272 were classified as being in a highly competitive situation, those between 0.272 and 0.377 were considered to be in a medium competitive situation, and cities with a CR5 above 0.377 (indicating that 37.7% of the financial market share is occupied by the five large state-owned banks) were classified as being in a low competitive situation.

Table 9 shows the results of the dynamic threshold regression model, with the level of urbanization included as a threshold variable. The coefficient of green credit was found to be −0.308 for cities below 45.1% urbanization and −0.355 in cities above 45.1% urbanization. It was found that the increase in the level of urbanization contributes to the low-carbon transition effect of green credit. Our findings confirm Hypothesis 3, namely, the low-carbon transition impact of green credit is heightened when urbanization levels reach 45.1%.

Table 9 indicates that as the market share of the five largest state-owned banks decreases, the dampening effect of green credits on carbon emission intensity subsequently increases (from −0.223 to −0.317). This finding supports Hypothesis 4 and also indicates that its policy effects act on emission reduction and energy saving through market-based mechanisms rather than administrative coercive orders.

### 4.6. Clustering Results

**Figure 5:**
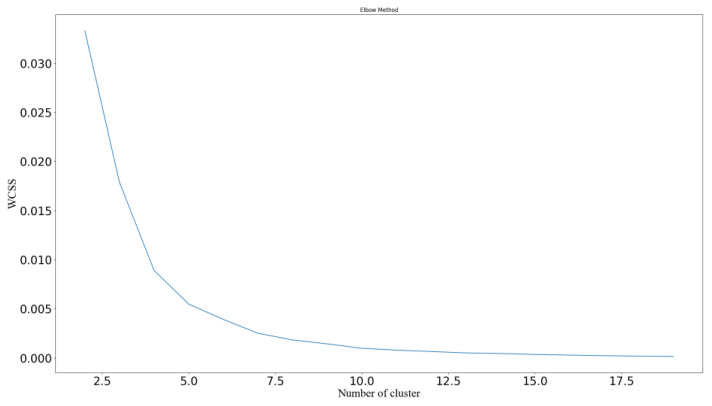
The results of the elbow method.

The elbow method based on Python 3.7.6 (as seen Figure 5) could not be used to determine the number of clusters, and there were inflection points at both 3 and 5. Following the method of Mohajer et al. [57], we found that Gap (5) was greater than Gap (3), and the results of the gap statistic showed that 5 was the right number of clusters.

**Figure 6:**
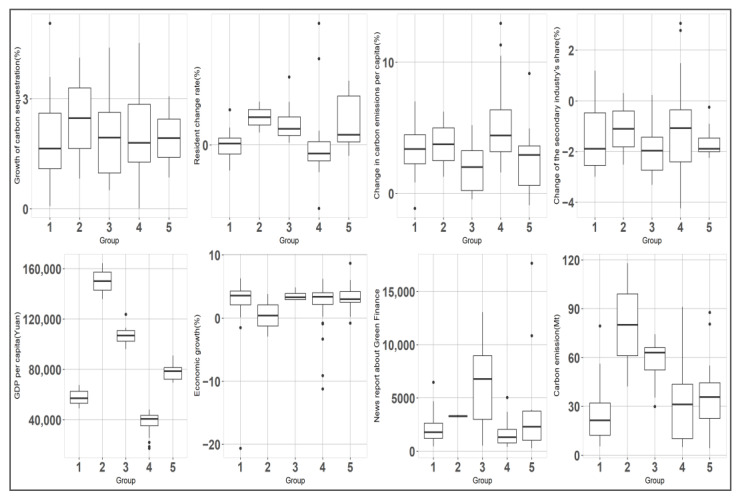
Key features of different categories of cities.

Based on the results of k-means clustering, we divided the development model of green credit in the Yellow River Basin into five types. Figure 6 shows the main characteristics of the different types of cities. This finding supports Hypothesis 5.

#### 4.6.1. Introduction to Different Types of Cities

**Figure 7:**
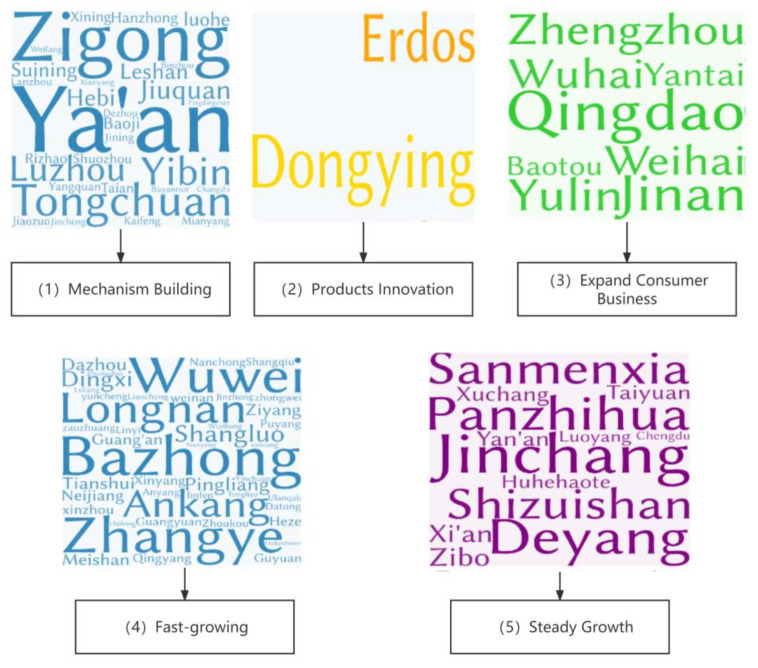
Green credit development patterns for cities in the Yellow River Basin.

Based on the characteristics of different categories of cities, we classified the green credit development pattern at the prefecture-level city level in the Yellow River Basin into five types (Figure 7).

The first type of green credit development pattern in the Yellow River Basin is mechanism building. We identified 30 cities of this type, including Ya’an, Hebi, Rizhao and Lanzhou (Figure 7). The average annual loss of residents in this type of city was found to be 0.2%, indicating that its industrial competitiveness is insufficient. The proportion of the output value of the secondary industry in such cities decreased by 1.4% on average from 2007 to 2020, and the process of de-industrialization was rapid. In 2020, the average total carbon emissions of such cities was 24.93 Mt, which was the lowest among all cities. On average, there were 2013 reports about the green finance in such cities in 2020, which means that those cities have a low level of development of green credit. Due to the low-carbon emission reduction pressure, these cities can focus on building green financial mechanisms such as credit constraint mechanisms and information disclosure mechanisms. The construction of these mechanism takes a long time, but they can be used to form a lasting, effective and healthy green financial system.

The second type of green credit development pattern in the Yellow River Basin is product innovation. This type of development was found to be suitable for two Yellow River basin cities: Dongying and Ordos. Both cities are resource-dependent major oil and coal producers in China, and they have the highest per capita gross domestic product (150,148.5 yuan). Despite the fact that these two cities were found to have the highest growth rates of carbon sequestration among all cities (2.4%), their total carbon emissions (80.1 Mt) were shown to be far greater than those of other cities. The development process of such cities is inextricably linked to the exploitation of resources. Therefore, the design of green credit policies for these two cities should focus on creatively transforming extractive industries into low-carbon industries.

The third type of green credit development pattern in the Yellow River Basin is consumer business expansion. This type of development was found to be appropriate for eight cities in this region (Jinan, Qingdao, Zhengzhou, Baotou, Yantai, Wuhai, Weihai, and Yulin). The majority of these cities are established industrial cities and provincial administrative centers. Strong demographic appeal, a local population growth of 1.4% per year, and a developed economy (GDP per capita of 107,651.1 yuan) are characteristics they share. In addition, these cities have a first-mover advantage in the development of green finance, with 6474 major media reports on their green finance development in 2020, well ahead of other types of cities. Therefore, based on their robust economic strength and extensive experience in implementing green credit, the green credit business for enterprises in such cities has matured and should focus on utilizing green credit tools to guide individuals’ green consumption habits in the future.

The fourth type of green credit development pattern in the Yellow River Basin is fast growth. This type of development was found to be appropriate for 44 cities in this region and encompassed the greatest number of cities of all categories, including cities such as Tianshui, Zaozhuang, Shangqiu, Heze, Zhumadian, and Hulunbuir. These cities have the greatest need to develop green credit for the reasons listed below. First, these cities are losing about 0.2% of their population annually and have the highest rate of growth in carbon emissions per capita of all types at 5%. Second, these cities have a per capita GDP of 38,748.89 yuan, which is significantly lower than that of other cities. Third, the level of green finance development in cities of this type was found to be the lowest. In conclusion, these cities cannot achieve carbon peaking and carbon neutrality goals on their own, and it is necessary to provide effective and concise green credit policy counseling and support to the local financial systems as soon as possible in order to bring about the positive effect of green credit in reducing the intensity of carbon emissions.

The fifth type of green credit development pattern in the Yellow River Basin is steady growth. This development pattern was found to be appropriate for 14 Yellow River basin cities, including Xuchang, Taiyuan, Chengdu, and Xi’an. They were found to have stable population growth, slow per capita carbon emission growth, and significantly lower total carbon emissions (37.6 Mt) in 2020 than cities in the second (80.1 Mt) and third types (57.5 Mt). With 3,737 relevant reports in 2020, the development of green finance in this category of cities is slower than in the third category of cities. If these cities adhere to the current green credit development pattern, it is anticipated that they will achieve the double carbon goal.

#### 4.6.2. Spatial Characteristics of the Green Credit Development Pattern

**Figure 8:**
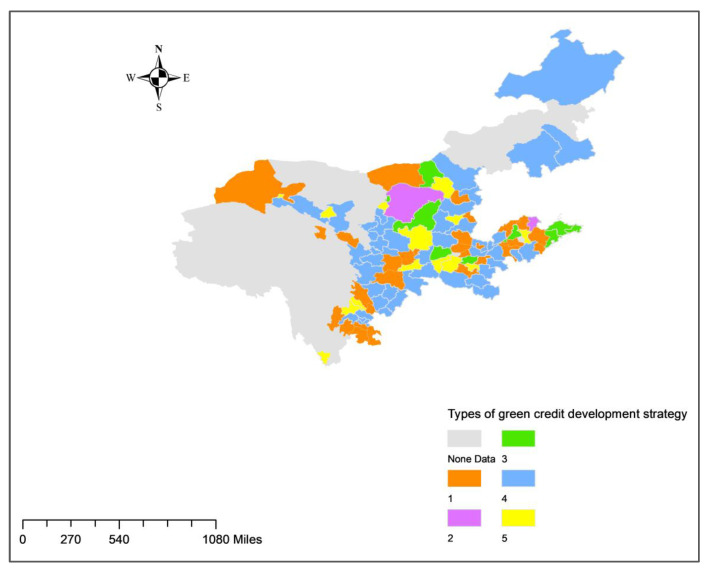
Green credit development pattern distribution.

In terms of the distribution of different development patterns, there was a clear agglomeration of cities in the first, third, fourth, and fifth types (Figure 8).

There were four agglomerations in the first category of cities. The first one appeared in Shandong and consisted of cities such as Dezhou, Jining, and Binzhou. The second agglomeration appeared in the northern part of Henan Province and consisted of cities such as Hebi, Kaifeng, and Jiaozuo. The third agglomeration appeared in the southern part of Sichuan Province and consisted of Yibin, Luzhou, Zigong, and Leshan. The fourth agglomeration appeared in the central part of Shaanxi Province, west of Xi’an, and consisted of the cities of Xianyang, Baoji, Hanzhong, and Tongchuan.

The development pattern of the third group of cities is to expand consumer businesses, three of which were found to be concentrated in the eastern part of the Shandong Peninsula, namely Qingdao, Yantai, and Weihai.

Rapid growth is the fourth type of green credit development pattern, with 44 cities falling into this category. They were found to be primarily concentrated in two regions. The first region was Linyi, Jining, and Heze in Southeastern Shandong Province plus Zhoukou, Nanyang, Zhumadian and Xinyang in Southern Henan Province. The second region was shown to span 15 cities in the provinces of Ningxia, Gansu, Shaanxi, and Sichuan, from Wuzhong in Ningxia in the north to Dazhou in Sichuan in the south. Additionally, numerous cities in Northeastern Inner Mongolia, Northwestern Gansu Province, and Eastern Shanxi Province were found to be suitable for this development pattern.

The pattern of development for green credit in the fifth type of city is steady growth. There was no significant agglomeration in this type of city. Additionally, this type of city appeared near the agglomeration of the fourth type of city, indicating that these cities perform better in terms of green credit and carbon emission control than surrounding cities. Jinchang in Gansu Province, Yan’an in Shaanxi Province, and Deyang and Chengdu in Sichuan Province are some examples.

## 5. Results Discussion and Policy Suggestions

### 5.1. Results Discussion

Our empirical regression results support Hypothesis 1, which posits that green credit helps control carbon intensity in the Yellow River Basin, indicating that green credit policy support is necessary to achieve dual carbon goals. This research conclusion is in line with the findings of Zheng et al. [63] and provides a basis for continuing to increase green credit investments in the future. Based on our regional heterogeneity analysis of the Yellow River Basin, the empirical findings of this paper also support Hypothesis 2.

Furthermore, our study identified a threshold effect of urbanization level on the low-carbon transition effect of green credit, which supports the results of a study by Song et al. [64], who argued that different levels of urbanization require exploring different low-carbon transition paths. Additionally, research on the threshold effect between the degree of bank competition and the effect of green credit low-carbon transition indicates the need to increase the level of competition in the banking sector, which is consistent with the findings of Zhao et al. [38]. Thus, the above findings support Hypotheses 3 and 4.

The results of the elbow and gap statistic methods indicated that the prefecture-level cities in the Yellow River basin can be classified into five categories for the development of green credit schemes. The k-means clustering algorithm was used to complete the clustering analysis based on the dynamic indicator changes and static indicator scales of each city. These results also supported Hypothesis 5, namely, green credit in the Yellow River Basin requires differentiated development strategies. In addition, the urban green credit development pattern classification based on clustering algorithm was different from the traditional entropy classification [65], so it can provide a new perspective for the planning and development of cities in the Yellow River basin. We developed five different green credit development strategies based on the different economic and social characteristics, carbon emission characteristics, and green financial development levels of the five types of cities. Instead of imitating the policies of neighboring cities, cities in different categories will implement green credit development strategies that possess different focuses, which will improve the overall efficiency of green credit operations in the Yellow River Basin.

### 5.2. Policy Suggestions

Based on the findings of our empirical study, the following suggestions are made for Yellow River Basin cities with various types of green credit development patterns.

Cities whose green credit development pattern is mechanism building are still in the early stages of green credit development. Since the pressure of carbon emission reduction is much lower in this type of city, there is ample time to build the basic mechanism of green credit. Green credit requires that the bank take the information related to the project and its operating company as the inspection standard in the process of making a loan decision [66]. Currently, the disclosure of environmental information by Chinese companies is insufficiently transparent, and some companies do not pay attention to ESG scores, which renders the lending criteria for green credit unclear and prevents green credit from effectively reducing carbon intensity. Consequently, in the early stages of green credit development, these cities should establish a dependable mechanism to make the entire green credit supply chain efficient and transparent. Local governments may, for instance, implement specific CO_2_ emission measurement standards and require local financial institutions and city-investment groups to use carbon emissions as a reference standard for credit granting and investment. In that case, they could avoid granting green credit to non-compliant enterprises.

Cities whose green credit development pattern is product innovation heavily rely on the extraction of fossil fuels. The experience of establishing green credit policies in other cities cannot be applied, because blindly limiting CO_2_ emissions can severely impede the economic development of such cities. China’s mining conditions determine that the current mining industry is dominated by small enterprises and is relatively fragmented. Some advanced equipment and technologies are difficult to apply in small- and medium-sized mining enterprises due to poor mining technology and equipment, as well as short industrial and capital chains. As a result, mining enterprises require financial support from green credit. China has launched the “National Green Mine List”. Some financial institutions, such as Jinshang Bank, have implemented a green credit policy to support green mining. Following the pattern of Green Mine Green Credit, these cities can rank the CO_2_ emission levels of energy-intensive enterprises in their jurisdictions. Companies that meet the carbon reduction requirements will be placed on the Green Pioneer list, and financial institutions will lower interest rates and increase credit limits. To achieve the above plan, the government and financial institutions need to innovate in enterprise carbon footprint tracking.

Cities with a high GDP per capita and a high level of green financial development (the third type) are able to introduce green consumer loans for individuals as an essential part of their green credit development pattern. In China, the carbon emissions generated by residents’ consumption account for about 53% of the total carbon emissions. In light of the fact that consumer spending is currently the primary factor propelling China’s economic expansion, it is crucial that the country raise public consciousness about the importance of conservation while simultaneously encouraging the green and low-carbon transformation of consumer behavior. Developed countries have launched a series of green consumer loans for individuals. For example, Barclays Bank in the UK has launched a “green home mortgage” to encourage customers to achieve an A or B predictive energy assessment (PEA) energy efficiency rating for their homes. Citibank in the U.S. offers residents quick access to financing for the purchase and installation of residential solar technology. Financial institutions in those cities can establish a personal carbon footprint and carbon account system by fully utilizing big data and other information technology means, thus providing a relatively comprehensive portrayal of consumers’ carbon consumption and carbon reduction behavior and providing basic data for the scientific and accurate quantification of carbon reduction. In addition, green credit products, such as lower interest rates for customers who purchase electric cars, can be introduced to guide residents toward adopting low-carbon living habits.

The cities with the development pattern of fast growth were found to have the lowest levels of green financial development, the fastest-growing per capita carbon emissions, and shrinking populations. Therefore, such cities have the greatest pressure to reduce carbon emissions and the greatest demand for green credits. Most of the cities in this group are experiencing economic downturns, so when banks decide where to implement green credits, it is more important to think about the region’s environmental benefits than the interest income that would be generated. Specifically, we think that the following three programs can be used to rapidly develop local green credit. First, the trend of population exodus will be long-term in this type of city due to the lack of competitive industries, and the area of built-up land will not increase. Therefore, green credit can support local afforestation and urban greening projects, as well as contribute to carbon neutrality from the perspective of increasing carbon sinks. Secondly, this type of city has the lowest level of green finance development, and local financial institutions and government agencies should help personnel learn about green credit policies and seek financial support from the provincial government. Finally, due to the poor economic level of this type of city, the green credit business of the same type of city in a region can be handled by one policy bank to reduce the running cost of green credit. Taking Sichuan Province as an example, Guang’an, Neijiang, Ziyang and Dazhou (which all belong to the fourth type) can be designated as one green credit business area, and the China Development Bank can be responsible for the green credit business in all four cities.

For cities whose green credit development pattern is steady growth, their populations are steadily increasing, their growth rate of carbon emissions is currently under control, and their level of green finance development is high. Therefore, such cities are in the process of transitioning to the third type, and their green credits should continue their previous strategies with modest adjustments based on future carbon emission trends.

## 6. Conclusions

This study was aimed to investigate the effectiveness of the green credit policy implemented in the Yellow River Basin in reducing local carbon emission intensity and to provide specific suggestions for the development pattern of green credit in each city. Through the application of multiple regression, spatial regression, and threshold regression models, the efficacy of green credit in reducing carbon emission intensity was verified while providing data support for the further expansion of green credit in the region. Moreover, based on the k-means clustering algorithm, 98 prefecture-level cities in the Yellow River Basin were classified into five categories of green credit development patterns considering both static and dynamic indicators.

The findings highlight the importance of considering regional heterogeneity and threshold effects when designing and implementing green credit policies. Additionally, the approach proposed for developing green credit patterns for prefecture-level cities can be extended to other regions and used as a reference for policymakers to promote low-carbon transitions in the financial sector.

The development patterns proposed in this study for the Yellow River Basin could serve as a reference for other regions and countries with similar characteristics, but adjustments should be made according to local realities and resource endowments. However, the general concept of using green credit as a tool to promote low-carbon transition and reduce carbon emissions can be more broadly applied and could be a viable option for other developing countries with limited credit resources.

According to the findings of this paper, the k-means clustering method can be applied to the analysis of regional green credit development patterns and has good explanatory power for the clustering results. However, this method produces unstable results for unbalanced samples and samples with outliers, which places high demands on the cleaning of the dataset and requires special attention when using large datasets for research. The main limitation of this paper is that the validity of the clustering results and related suggestions were based on existing data. More reasonable indicators can be built to contribute to the calculation of clustering algorithms in the future as carbon emission-related data transparency increases and green credit policies are refined.

## Figures and Tables

**Table 1:** Key factors affecting low-carbon transition.

Influencing Factors	Description	Reference
Awareness of environmental protection	Government awareness of environmental regulation contributes to low-carbon transition.	[24]
The size of a city’s population	Large-scale population movements can affect carbon emission characteristics.	[25,26]
Education level	Rising education levels contribute to low-carbon transition.	[27]
Industrial structure	Manufacturing-dominated regions tend to have a higher scale of carbon emissions.	[28]
Foreign direct investment	Some FDI will promote a local low-carbon transition while others are looking for “pollution havens”.	[29,30]
Economic potential (growth).	The environmental Kuznets curve theorizes an inverted U-shaped relationship between economic growth and carbon emissions.	[26,31]

**Table 2:** Design of sustainable development patterns in previous studies.

Framework Type	Number of Indicators	Reference
Sustainable finance	15	[40]
Sustainable finance assessment	63	[41]
Sustainable finance risk index	22	[43]

**Table 3:** Explanations for variables in regressions.

Variables		Code	Note	Unit
Explained variable	Carbon intensity	Carbon	Carbon emission/GDP	(Mt/Billion yuan)
Explanatory variable	Green credit	GreenC	Total interest expenses minus interest expenses of “six high” industries ^1^	100 million yuan
Control variables	Environmental protection	EP	The proportion of words related to environmental protection in the government work report to all words	%
	Resident population	PEO	Population regularly residing in a city	10,000
	Education level	EL	Share of population with higher education	%
	Industrial structure	IS	Share of secondary industry output in GDP	%
	Growth	Growth	GDP growth rate	%
	Degree of openness	FDI	Foreign direct investment	$10,000
Substitution of explanatory variable	Energy intensity ^2^	Energy	Total energy consumption/GDP	(Mt/Million yuan)

^1^ Six high industries: chemical, oil, electricity and heat, ferrous metal smelting, non-ferrous metal smelting, and non-metal production. ^2^ All energy consumption is converted to standard coal.

**Table 4:** Descriptive statistics for variables.

Variable	Obs	Mean	Std. Dev.	Min	Max
GreenC	1485	15.09	20.32	0.2648	152.911
Carbon	1484	0.2557	0.1749	0.0349	1.1492
EP	1485	0.0033031	0.0014271	0	0.011931
PEO	1485	397.0676	258.9664	19.37	2095
HR	1485	1.554535	2.015629	0.003865	12.7643
IS	1485	49.91055	11.56516	15.6	84.39
GROWTH	1485	10.19219	4.391496	−20.63	25.8
FDI	1485	43,845.25	116,680.8	0	1,300,000

**Table 5:** Clustering indicators.

Type	Indicators	Specific Data	Reference Year
Static	Development level	GDP per capita	2020
	Economic growth	The growth of GDP	2020
	Carbon emission level	Carbon emissions	2020
	Green finance development	The news report about local green finance	2020
Dynamic	Industrial structure change	The average change in the proportion of secondary industry output value	2006–2020
	Development potential	The average change of residents	2006–2020
	Carbon sequestration growth	The average change of carbon sequestration	2006–2020
	Resistance to emission reduction	The average change of carbon emissions per capita	2006–2020

**Table 6:** Benchmark regression results.

	(a)	(b)	(c)
Variables	Carbon	Carbon	Carbon
GreenC	−0.378 *** (−59.58)	−0.020 * (−1.69)	−0.040 * (−1.83)
EP		0.011 (−0.92)	0.016 (−1.13)
PEO		−0.326 *** (−7.73)	0.079 (−1.33)
EL		−0.616 *** (−35.07)	0.074 *** (−6.29)
IS		0.040 *** (−4.05)	−0.371 *** (−10.51)
Growth			−0.030 *** (−3.35)
FDI			−0.001 (−0.86)
Individual effect	No	No	Yes
Time effect	No	No	Yes
Control variables	No	Yes	Yes
Constant	−0.796 *** (−14.18)	6.820 *** (−21.07)	−0.259 (−0.66)
Obs	1470	1470	1470
R^2^	0.1911	0.2726	0.9593

Notes: z-statistics in parentheses; *** *p* < 0.01, * *p* < 0.1.

**Table 7:** Regression results for robustness.

Variables	GMM	Durbin Model	Robustness Check		
			Energy Intensity	Digitalization	Policy Impact
GreenC	−0.338 ** (−35.05)	−0.067 ** (−1.97)	−0.372 *** (−39.24)	−0.041 * (−1.85)	−0.351 *** (−18.40)
EP	0.015 (1.12)	0.026 ** (2.04)	−0.014 (−0.81)	0.168 (1.19)	−0.008 (−0.36)
PEO	−0.048 (−0.97)	−0.100 * (−1.91)	−0.511 *** (−6.87)	0.064 (1.07)	−0.612 *** (−6.86)
EL	−0.027 *** (−2.05)	0.072 *** (7.06)	−0.083 *** (−5.92)	0.076 *** (6.47)	−0.196 *** (−12.19)
IS	0.199 *** (5.58)	−0.352 *** (−10.70)	−0.180 *** (−5.00)	−0.368 *** (−10.45)	0.274 *** (6.05)
Growth	0.059 *** (5.79)	−0.027 *** (−3.54)	0.029 *** (2.80)	−0.029 *** (−3.34)	0.123 *** (9.82)
FDI	−0.003 (−1.63)	0.000 (0.08)	−0.006 *** (−3.53)	−0.001 (−0.98)	−0.006 *** (−2.77)
Internet				−0.028 ** (−2.25)	
R^2^	0.259	0.1906	0.9602	0.9600	0.8959

Notes: z-statistics in parentheses; *** *p* < 0.01, ** *p* < 0.05, * *p* < 0.1.

**Table 8:** Regression results for regional heterogeneity.

Variables	Upper	Middle	Lower
GreenC	−0.101 * (−1.96)	−0.141 *** (−3.78)	−0.376 *** (−6.16)
Individual effect	Yes	Yes	Yes
Time effect	Yes	Yes	Yes
Control variables	Yes	Yes	Yes
R^2^	0.9642	0.9669	0.9464

Notes: z-statistics in parentheses; *** *p* < 0.01, * *p* < 0.1.

**Table 9:** Regression results for threshold heterogeneity.

Variables	Low Urbanization	High Urbanization	Low Competition	Medium Competition	High Competition
GreenC	−0.308 *** (−29.40)	−0.355 *** (−39.25)	−0.223 * (−10.87)	−0.260 *** (−13.80)	−0.317 *** (−16.61)
Individual effect	Yes	Yes	Yes	Yes	Yes
Time effect	Yes	Yes	Yes	Yes	Yes
Control Variables	Yes	Yes	Yes	Yes	Yes
R^2^	0.207	0.207	0.198	0.198	0.198

Notes: z-statistics in parentheses; *** *p* < 0.01, * *p* < 0.1.

## Data Availability

CEADs: https://www.ceads.net.cn/; China City Statistical Yearbook: https://data.cnki.net/v3/Yearbook/Single/N2022040095.
